# AmbuFlex: tele-patient-reported outcomes (telePRO) as the basis for follow-up in chronic and malignant diseases

**DOI:** 10.1007/s11136-015-1207-0

**Published:** 2016-01-20

**Authors:** Liv Marit Valen Schougaard, Louise Pape Larsen, Anne Jessen, Per Sidenius, Liv Dorflinger, Annette de Thurah, Niels Henrik Hjollund

**Affiliations:** AmbuFlex/WestChronic, Regional Hospital West Jutland, Herning, Denmark; Department of Neurology, Aarhus University Hospital, Aarhus, Denmark; The Danish Cancer Society, Copenhagen, Denmark; Department of Rheumatology, Aarhus University Hospital, Aarhus, Denmark; Department of Clinical Medicine, Aarhus University, Aarhus, Denmark; Department of Clinical Epidemiology, Aarhus University Hospital, Aarhus, Denmark

**Keywords:** Patient-reported outcomes, telePRO, ePRO, Clinical practice, Outpatient clinic, Outpatient follow-up

## Abstract

**Purpose:**

A tele-patient-reported outcome (telePRO) model includes outpatients’ reports of symptoms and health status from home before or instead of visiting the outpatient clinic. In the generic PRO system, AmbuFlex, telePRO is used to decide whether a patient needs an outpatient visit and is thus a tool for better symptom assessment, more patient-centred care, and more efficient use of resources. Specific PROs are developed for each patient group. In this paper we describe our experiences with large-scale implementations of telePRO as the basis for follow-up in chronic and malignant diseases using the generic PRO system AmbuFlex.

**Methods:**

The AmbuFlex concept consists of three generic elements: PRO data collection, PRO-based automated decision algorithm, and PRO-based graphical overview for clinical decision support. Experiences were described with respect to these elements.

**Results:**

By December 2015, AmbuFlex was implemented in nine diagnostic groups in Denmark. A total of 13,135 outpatients from 15 clinics have been individually referred. From epilepsy clinics, about 70 % of all their outpatients were referred. The response rates for the initial questionnaire were 81–98 %. Of 8256 telePRO-based contacts from epilepsy outpatients, up to 48 % were handled without other contact than the PRO assessment. Clinicians as well as patients reported high satisfaction with the system.

**Conclusion:**

The results indicate that telePRO is feasible and may be recommended as the platform for follow-up in several patient groups with chronic and malignant diseases and with many consecutive outpatient contacts.

## Background

The use of patient-reported outcomes (PRO) in clinical practice is becoming increasingly common, and several studies have reported improved patient–clinician communication, early recognition of important symptoms, more effective self-management, and better use of resources [1–7]. A PRO measure is the patient’s own report of health status, e.g. symptoms, health-related quality of life, and functional status. The American Food and Drug Agency defines PRO as “A measurement based on a report that comes directly from the patient about the status of a patient’s health condition without interpretation of the patient’s response by a clinician or anyone else” [8]. This definition focuses on the source of information and emphasises the importance of the patient perspective.

Experiences of use of electronic PRO (ePRO) systems have been extensively reported [1–7, 9–12]. The primary goal is to improve quality of care by better support of clinical activities, e.g. prospectively symptom monitoring used as consultation support. Haverman et al. [13] describe an ePRO system in daily paediatric clinical practice developed to systematically monitor health-related quality of life (HRQOL) in children with a chronic arthritic disease, and Snyder et al. [14] describe the development of the website PatientViewpoint, designed to collect PRO in outpatient clinical oncology. Both systems use PRO before a scheduled consultation, but Snyder et al. [14] emphasise the potential of using PRO between visits.

Integrating PRO into clinical practice has great potential when PRO becomes a central part of the patient pathway and is fully incorporated into daily clinical practice [15]. Follow-up for patients with chronic diseases is traditionally lifelong and managed by regular pre-scheduled visits. These visits may occur when the patient is well, and neither the patient nor the clinician finds the visit necessary [16]. Unnecessary outpatient visits place an increasing burden on already overstretched healthcare services, making it difficult to respond rapidly to a patient’s acute requests for attention [16, 17]. A PRO assessment can be used to evaluate the need for a clinical visit, thereby managing resources better [18]. Outpatient clinics could potentially minimise large numbers of routine visits if a PRO assessment is obtained when the patient is still at home. The benefit comes from making PRO the basis for outpatient follow-up instead of now—the patient visit [15]. However, few attempts, if any, have been made to make PRO the basis for outpatient follow-up [15].

In ePRO systems, PRO data may be collected at home or in the waiting room using computers, tablets or a patient kiosk. If PRO is used as the basis for outpatient follow-up—and even a replacement of unnecessary visits, PRO data must obviously be collected at a distance (e.g. from home), and we will refer to the latter as telePRO [18].

AmbuFlex is a generic clinical telePRO system for mixed-mode (web and paper) PRO data collection for use in clinical practice. The overall goal is to use PRO for clinical decision support to improve quality of care, promote patient-centred care, optimise the use of resources in the healthcare system, and use data for research purpose (Table 1).

**Table 1:** General aims in telePRO projects

Improve quality of care by flagging important symptoms and produce better documentation of patient information
Promote patient-centred care with focus on patients’ needs and knowledge about own disease
Optimise the use of resources in the healthcare system
Use PRO data in research and hospital quality assurance

The aim of this paper was to describe experiences with implementing telePRO as the basis for follow-up in chronic and malignant diseases using the generic PRO system AmbuFlex, where the patients define the need of an outpatient consultation by delivering PRO.

## Methods

AmbuFlex is the frontend of the WestChronic system, used for research purposes in clinical epidemiological studies since 2004 [18]. AmbuFlex consists of three generic, configurable elements (Table 2) [18]: PRO data collection, PRO-based automated decision algorithm, and PRO-based graphical overview for clinical decision support. We describe our experiences with respect to these three elements.

**Table 2:** Elements of clinical application of tele-patient-reported outcomes (telePRO) [18]

A. PRO data collection	Questionnaire and pilot tests
	Referral
	Data collection modes
B. PRO-based automated decision algorithm	Thresholds defined by published cut-off values
	Thresholds defined by clinicians
C. PRO-based graphical overview for clinical decision support	Course-oriented graphic overview
	Configuration of PRO for clinical decision support

### PRO data collection

The development of the disease-specific PRO questionnaire is fundamental for the validity, reliability, and acceptability to patients and clinicians [19]. It is vital that the questionnaire reflects clinically relevant aspects of the actual clinical situation. Clinicians as well as patients must find all items in the questionnaire relevant. Face validity was ensured during the development process for each new patient group. If clinically relevant validated instruments were not available, we developed ad hoc items if necessary. This process included inputs from specialists in the disease area, a review of literature, and an interview with patients [20]. We only developed ad hoc items, not scales. Pilot tests of questionnaires were conducted to identity potential problems such as low relevance of items, ambiguity of items, and lack of important issues [21, 22]. The AmbuFlex system automatically prompts patients by letter or e-mail to answer the questionnaire either online or in paper form at a scheduled time, and therefore referral is a prerequisite. TelePRO referral is managed as part of daily clinical practice and decided by the individual clinician based on patient characteristics and his or her experiences and preferences. Information on the mixed-mode data collection (web-based, paper-based, or mixed-mode) and other logistics considerations related to administration, such as reminders and data import and export are reported elsewhere [18].

### PRO-based automated decision algorithm

AmbuFlex is designed to make automated decisions, in which a PRO assessment is used to divide patients into two categories: those who need clinical attention and those who do not based on defined algorithms and thresholds. Two different approaches were used: external cut-off values based on validated PRO instruments or thresholds defined by clinicians where each response category was assigned a colour code. When a threshold was defined by clinicians, the goal was to have a false-negative rate close to zero, whereas the rate of false positive was of less concern [18]. A clinical expert group divided each response categories into three levels: green, yellow, or red. These assignments were entered into the server software’s configuration utility for each specific questionnaire. Based on the incoming PRO data, the server algorithms would consecutively categorise the patients’ present state. If all responses had a green code, it would signal that no contact was needed; if one or more responses had a red code, the patient must be seen or contacted; while a yellow code indicated that the patient may need to be contacted and a clinician should make the decision based on the PRO overview (cf. below). The AmbuFlex system keeps track of patients with red and yellow status and non-responders, who are presented to the clinicians on an alert list.

### PRO-based graphical overview for clinical decision support

A clinical PRO system should enable the clinician to access systematically collected PRO data to support monitoring and clinical decision-making [1, 19]. AmbuFlex uses the PRO data to display the course of symptoms and prioritises issues by flagging symptoms that need further attention. A graphical overview presented to the clinician can guide clinical decisions. A graphical PRO overview interface was developed, and a configuration utility enables adaption to the specific patient group.

## Results

As of December 2015, AmbuFlex has been implemented in nine diagnostic groups in Denmark. This paper included seven clinical projects with internal project management by AmbuFlex funded by Central Denmark Region, one randomised controlled trial with external project management funded by Aarhus University Hospital, and one clinical project with external project management funded by the Danish Cancer Society. The characteristics of all projects are presented in Table 3. In addition to the general aims in Table 1, each implementation had additional aims according to the specific diagnostic group. In epilepsy (b), narcolepsy (c), sleep apnoea (e), prostate cancer (f), asthma (h), and renal failure (i), the aim was to facilitate greater flexibility in the provision of care and thereby increase patient self-management, improve the quality of care, and achieve a better utilisation of resources. In rheumatoid arthritis (d), the primary aim was to examine the effect of a PRO-based telemedicine intervention to assess flare-ups in disease activity using a validated PRO instrument [23, 24] combined with a blood test. In colorectal cancer (g), one of the aims was to use PRO data to assess a patient’s health status before chemotherapy treatment in order to prescribe the chemotherapy in advance. In coronary heart disease (a), the primary aim was to screen patients for depression and anxiety.

**Table 3:** Characteristics of nine AmbuFlex projects using telePRO in clinical practice, December 2015

Diagnostic group	In operation from	Mode	Elements (cf. Table 2)	PRO instruments	Outpatient clinics	Patients referred	Patients referred pr. mo. (average the last 6 mo.)	Reminders	Initial response rate %	Response rate follow-up %
a. Heart disease	July 2011	Paper/web	A, B	HADS	n/a	4432	106	1	81	No follow-up
b. Epilepsy	March 2012	Paper/web	A, B, C	WHO-5, SF-36, SCL-92, ad hoc items	3	4214	43	3	92	95
c. Narcolepsy	Sept. 2013	Paper/web	A, B, C	WHO-5, SF-36, ESS, SCL-92, ad hoc items	2	70	1	3	98	93
d. Rheumatoid arthritis	May 2014	Paper/web	A, B, C	FLARE, HAQ	2	300	n/a	0	93	98
e. Sleep apnoea	July 2014	Paper/web	A, B, C	ESS, SCL-92, ad hoc items	2	2822	272	3	95	97
f. Prostate cancer	Sept. 2014	Paper/web	A, B, C	EORTC QLQ-C30, DANPSS, IIEF-5, RT-ARD, WHO-5	3	347	28	2	98	95
g. Colorectal cancer	March 2015	Web	A, B, C	Ad hoc items	1	477	60	0	n/a	n/a
h. Asthma	April 2015	Web/app	A, B, C	ACQ	1	25	2	1	91	n/a
i. Renal failure	Aug. 2015	Paper/web	A, B, C	HADS, SCL-92, EQ-5D, BIPQ, ad hoc items	1	448	90	1	84	90

In heart disease (a), the number of outpatient clinics is not relevant as follow-up takes place in general practice. In rheumatoid arthritis (d), inclusion was completed in July 2015. In colorectal cancer (g), no referral takes place and the denominator is undefined. In asthma (h), a follow-up response rate cannot yet be calculated. (d) External project management and funded by Department of Rheumatology, Aarhus University Hospital, Denmark. (f) External project management and funded by The Danish Cancer Society, Copenhagen, Denmark. (a), (b), (c), (e), (g), (h), (i) Internal project management by AmbuFlex and funded by Central Denmark Region, Denmark

*Abbreviations n/a* not applicable, *HADS* Hospital Anxiety and Depression Scale [25], *WHO*-*5* WHO-Five Well-Being Index [26], *SF*-*36* Short Form 36 Health Survey [27], *SCL*-*92* Symptom Checklist 92 [28], *ESS* Epworth Sleepiness Scale [29, 30], *FLARE* Flare Assessment in Rheumatoid Arthritis questionnaire [23, 24], *HAQ* Health Assessment Questionnaire [31], EORTC QLQ-C30 [32], *DANPSS* The Danish Prostate Symptom Score [33], *IIEF*-*5* International Index of Erectile Function [34], *RT*-*ARD* Radiotherapy induced anorectal dysfunction [35], *ACQ* Asthma Control Questionnaire [36], EQ-5D [37], *BIPQ* Brief Illness Perception Questionnaire [38]

### PRO data collection

#### Questionnaire and pilot tests

The questionnaires were, whenever possible, based on validated PRO instruments, e.g. WHO-5 [26], SF-36 [27], HADS [25], EORTC QLQ-C30 [32]. Ad hoc items were developed in five projects (Table 3). All questionnaires were pilot-tested by the patients. For example, a total of 20 outpatients with epilepsy were included to pretest the epilepsy questionnaire. The majority of patients found the questionnaire easy to use apart from some problems due to recall and linguistic skills. They perceived the items as relevant and did not report any lack of important issues. The questionnaire provided important information specific to aspects of daily life with epilepsy. After a pilot test, the PRO application was implemented, and experiences with the questionnaire were continuously evaluated. Items were revised in an iterative process until saturation was reached after 2–4 months. After this period only few minor changes were usually needed [18].

#### Referral

All patients were individually referred, and a total of 13,135 outpatients from 15 clinics have been referred to telePRO follow-up. In epilepsy, it was estimated that 70 % of all outpatients were referred. Numbers of referred patients in each specific project by December 2015 are listed in Table 3. Criteria for referral differed between the diagnostic groups due to the use of different guidelines for monitoring the disease course. In epilepsy (b), narcolepsy (c), sleep apnoea (e), and prostate cancer (f), the patients were referred to AmbuFlex by the clinician, hence received questionnaires at pre-specified intervals (3, 6, or 12 months). Patients with rheumatoid arthritis (d) and asthma (h) were referred with a 3-month interval between questionnaires.

#### Data collection modes

PRO data were collected with mixed-mode (paper or web-based) in all projects except two, where only the web-based method was applied due to a tight time schedule [colorectal cancer (g) and asthma (h)]. Up to three reminders were applied (Table 3). A total of 18,912 questionnaires have been collected. The response rates for the initial questionnaire ranged from 81 to 98 % (Table 3). The highest rates were found among patient with prostate cancer (f) (98 %), narcolepsy (c) (98 %), and sleep apnoea (e) (95 %). The lowest rate was found among patients with heart disease (a) (81 %), and renal failure (i) (84 %), where only one reminder was used. During follow-up, the rates were between 90 and 98 %. The average proportion of web-based answers was 56.7 %.

### PRO-based automated decision algorithm

#### Thresholds defined by published cut-off values

This method was used in patients with heart disease (a), rheumatoid arthritis (d), and asthma (h). In rheumatoid arthritis (d), published cut-off values [23, 24] were used combined with objective data (blood test indicating inflammation) to indicate when the patient should be seen in the clinic. In patients with coronary heart disease (a), an automated algorithm based on published cut-off values divided patients into nine groups according to no, moderate, or severe symptoms on the two scales of anxiety and depression [25]. Based on these values, the AmbuFlex system automatically generated a personalised letter with screening results. If moderate or severe symptoms were present, the patient was advised to consult his general practitioner and bring along the letter [18].

#### Thresholds defined by clinicians

This method was used in patients with epilepsy (b), narcolepsy (c), sleep apnoea (e), prostate cancer (f), and colorectal cancer (g). In epilepsy (b), examples of red responses were self-reported aggravation of seizures or planning of pregnancy. Examples of yellow responses were self-reported presence of one or more symptoms (e.g. headache, dizziness and tremor) or social difficulties. Patients could in all cases request a contact and overrule any automated decision of “no contact” when answering the question “Which form of consultation do you feel would be most appropriate for you at this point in time?”. If their answer was “I would like the clinic to contact me” or “I would like to book an appointment”, the response was always determined to be red. Distribution of green, yellow, and red responses in epilepsy, sleep apnoea, prostate cancer, and heart disease is presented in Table 4. In epilepsy and sleep apnoea, 48 and 57 %, respectively, of the incoming PRO questionnaires could be handled with no other contact than the PRO. In epilepsy, the distribution is illustrated in Fig. 2.

**Table 4:** Distribution of PRO-based automated decisions and patient contact to the clinic in four telePRO projects, December 2015

Diagnostic group	Total PRO responses	Green responses (%)	Yellow responses (%)	Red responses (%)	No further contact to the clinic %	Contact to the clinic^a^ %
Epilepsy	8256	1035 (12)	5110 (62)	2111 (26)	48	52
Sleep apnoea	1424	202 (14)	673 (47)	549 (39)	57	43
Prostate cancer	347	38 (11)	128 (37)	181 (52)	26	74
Heart disease^b^	1335	932 (69.8)	0	403 (30.2)	n/a	n/a

^a^Contact to the clinic: a telephone consultation or a visit at the outpatient clinic

^b^Hospital Anxiety and Depression Scale (HADS) [25] in patients with cardiovascular disease 2011–2013. All patients received a letter with screening results. Patient with red status (moderate or severe symptoms) were advised to consult his general practitioner (GP) and bring along the letter. n/a: Data of contact to the GP is not available

### PRO-based graphical overview for clinical decision support

#### Course-oriented graphic overview

**Fig. 1:**
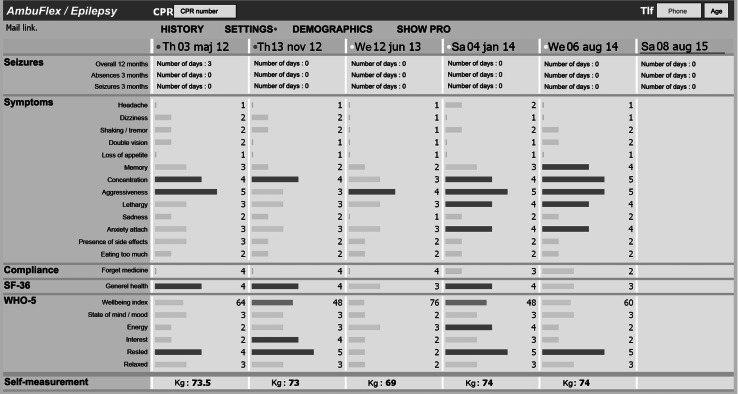
Screen capture of the clinicians’ overview in epilepsy clinics accessed from the Electronic Health Record of Central Denmark Region (MidtEPJ). The *colour codes* in the *upper row* indicate the result of the automated PRO algorithm (*red*: definite need of contact, *yellow*: possible need of contact, *green*: no need of contact). The *bars* indicate the severity of the symptom, e.g. a *red colour* indicates a self-reported problem. *Note*: Labels were translated from the Danish. (Color figure online)

A graphical overview of the PRO results over time was designed in each AmbuFlex implementation, and presented graphically to the clinician (Fig. 1). The overview was integrated via a link to the electronic health record system in 13 out of 15 outpatient clinics in one Danish region, whereas the other clinics accessed the database via an external secure webpage. Each vertical column in Fig. 1 represents a PRO questionnaire. The items and responses were displayed as a “pop-up tip” when the user puts the mouse icon over the displayed bar. Vertically, the overview presented the actual situation and horizontally the change in response over time [18].

#### Configuration of PRO for clinical decision support

The process of selecting items and grading severity was based on inputs from the clinicians. Colour codes were used to graduate the severity of symptoms or mark attention to a worsening problem. Some items did not fit into the overview, e.g. items with a yes/no response scale. All items however, were available when clicking at ‘Show PRO’, enabling the clinicians to see specific questionnaire responses. In that way, clinicians got a complete list of all questions and answers in the specific questionnaire with detailed information about the items and colour codes.

### Example: patient flow in outpatients with epilepsy

**Fig. 2:**
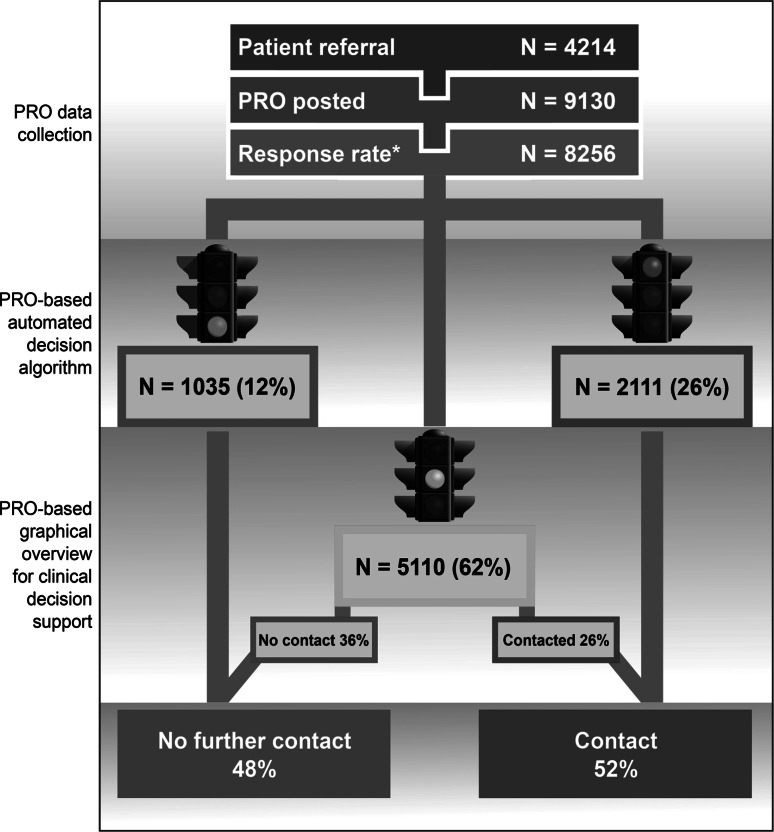
Flow chart for outpatients with epilepsy, December 2015. *Green* response: No need of contact. *Yellow* response: May need contact. A clinician has to decide whether further contact is needed. *Red* response: Definite need of contact or the patient asks for a consultation. *Estimated response rate with first questionnaire was 92 %. (Color figure online)

An overview of the patient flow for outpatients with epilepsy is shown in Fig. 2. In December 2015, about 70 % of the population of outpatients with epilepsy (b) in Central Denmark Region was referred to AmbuFlex (*N* = 4214). A total of 9130 questionnaires were posted and 8256 responses have returned. The response rate was estimated to be 92 % for the initial questionnaire and 95 % for the subsequent ones. Among the 8256 responses, the distribution was as follows: green (12 %), yellow (62 %), and red (26 %). In 38 % (green and red responses), the PRO-based automated algorithm decided automatically whether the patient should be seen or not. In the remaining 62 %, the clinician most often (36 %) decided that no further contact was needed. Overall, 48 % had no further contact than the PRO, while 52 % had a subsequent follow-up visit in the outpatient clinic or a telephone consultation.

### Feedback from clinicians and patients

The AmbuFlex implementation process for each patient group took place in one selected outpatient clinic (the index department). Experiences showed that the system was easily transferred to other outpatient clinics for the same patient group without modification or with only a few changes. Hence, an implementation seems to be specific for a patient group, not for the organisation [18]. The implementation process in epilepsy has been positively evaluated from a clinical as well as a patient perspective [39]. Both system and questionnaire have been developed in close cooperation with clinicians. Patients’ experiences of using PRO in clinical practice have been positive. Patients did not feel insecure with communication solely being written. Overall, the patients reported several advantages including greater flexibility in care, saving of time, improved information to the clinicians, increased knowledge about their own disease, and a good societal perspective in relation to sympathising with other patients’ needs [39].

## Discussion

So far, the generic PRO system AmbuFlex has been implemented in nine diagnostic groups at 15 outpatient clinics in Denmark. In these cases, telePRO was used as the basis for the contact between the patient and the clinic. In epilepsy clinics, up to 70 % of outpatients were referred to telePRO-based follow-up, and up to 48 % of the incoming PRO questionnaires could be handled with no other contact than the PRO.

Although results from other large-scale PRO implementations have been reported [40–42], we have not been able to identify other examples in which PRO was used *instead* of scheduled visits and as the basis for the contact. In nearly half of the cases, telePRO was the only contact between the patient and the clinic. This is important for two reasons. First, when telePRO is the basis for the contact, it is no longer an optional or added task for patients and clinicians, but fully integrated into the patient care. This being achieved, the other potential benefits of PRO measures in clinical practice may be obtained such as improved quality of care, better symptom assessment, more patient-centred care, and more efficient use of resources [7, 42, 43]. Second, when potentially up to half of the visits may be replaced by a less resource demanding activity, there may be an economic argument for the shift to telePRO, because the savings made could cover the expenses associated with implementation of telePRO. A recent national analysis of the clinical use of telePRO initiated by the Danish government and Danish regions based solely on experiences with AmbuFlex concluded that there was an overall economical potential related to (1) cancelled consultations, (2) reduced reimbursement of patient transportation costs, and (3) reduced need of destruction of medicine in cancer treatment. A national strategy for dissemination of clinical telePRO was therefore recommended [44].

Implementation of telePRO in clinical practice involves several issues related to the specific patient group, questionnaire, technology, and organisation [19, 41]. Unfortunately, a PRO instrument with documented psychometric properties relevant for the actual clinical decision is often not available, especially in non-malignant diseases. In other cases, a relevant PRO was available, but no relevant cut-off values were documented. In these cases, the content and cut-off values were negotiated based on iterative inputs from clinicians, review of the literature, and interviews with patients [18]. Reliability and other validity tests are of great value in improving data quality. Reliability studies of AmbuFlex questionnaire including developed ad hoc items will be conducted in the future. The key issues in all AmbuFlex implementations are involvement of patients as well as support from frontline clinicians and administrative leaders [18]. Since telePRO is used for clinical decisions, even automatic decisions, strict attention to the sensitivity should be given, and the algorithm should also reflect this. To ensure high sensitivity, clinicians assigned a green, yellow, or red colour to each item response in the questionnaires. More than about 15 % automatic *green* responses can rarely be reached without compromising the security in relation to detect patients in need of attention. All outpatients are instructed to contact the clinic in case of sudden worsening.

High response rates are needed to achieve satisfactory rates of completion [41]. In the Dutch ePRO system KLIK, an average of 70 % of patients have completed questionnaires prior to the consultation [45, 46], but in other systems only half of the patients completed the assessment before a visit [42]. Patients’ health literacy can be a barrier when completing PROs [41] and should be taken into account by the clinician before referring a patient to telePRO follow-up. If high response rates are crucial, a mixed-mode survey must be considered. Findings from randomised studies in other patient populations support this [47, 48]. We use a dual-mode system in which patients can choose between web and paper questionnaires that results in response rates beyond 90 %. This is discussed in more detail elsewhere [18].

Health professionals value PRO data when they are useful for the clinical decision-making process, whereas potential barriers may arise when the use of PRO appears to be disruptive to normal work flow [5, 49]. From the clinicians’ perspective, telePRO information should be of major importance in the clinical assessment of the disease, and a physical examination should not be central for evaluation of the patients’ clinical status [18]. The clinicians have to reallocate resources to handle the incoming questionnaires and have available time slots for consultations when an appointment is needed. A potential barrier in telePRO could be clinicians’ reluctance to an open-access strategy, as clinicians may believe that most patients would want to be seen in the outpatient clinic. This is, however, not the case in outpatients with epilepsy, as only 23 % ask for a consultation. Another barrier related to successful implementation is clinicians’ lack of knowledge on how to effectively utilise PRO data in their clinical practice [45, 49]. Santana et al. [45] recommend learning programmes teaching clinicians how to use and react to PRO in clinical practice. We work closely with frontline clinicians who are motivated; however, it can be a challenging process to convince a medical staff who is not enthusiastic about the use of PRO in clinical practice. Training programmes could be useful in telePRO implementations increasing involvement and motivation in the entire healthcare team.

Based on our experiences so far, we can suggest some characteristics that should be fulfilled when considering a patient group for telePRO. First, patients should need regular and systematic disease monitoring with several outpatient follow-up. Second, the disease activity and thus the need of medical attention should vary over time. Third, PRO should be essential for the clinical evaluation, if necessary, together with laboratory data in the health record, and finally, the evaluation of the health status should obviously not depend on a physical examination, e.g. auscultation (Table 5). We believe that a substantial proportion of diagnostic groups in outpatient follow-up fulfil these criteria.

**Table 5:** Characteristics of patient groups recommended for telePRO implementations

Repeated outpatient follow-up visits
Fluctuation in disease activity
PRO essential for clinical evaluation
No need for physical examination

There is a great potential in engaging patients more extensively in PRO data collection and implementation. For outpatients with epilepsy, we have now designed a website, “My Epilepsy”, where patients have access to their own questionnaire responses, and contacts to the outpatient clinic can be initiated by the patient based on a PRO assessment. The website is linked with the Danish National Health Website (‘Sundhed.dk’). A randomised controlled study comparing the actual AmbuFlex with this new open-access version is in progress. Based on the results, this patient-centred follow-up approach will probably be used extensively in future telePRO implementations.

## Conclusion

To our knowledge, AmbuFlex is the first generic PRO system that has transferred follow-up of entire diagnostic groups to a PRO platform for outpatient care. The AmbuFlex system is generic and not limited to specific diagnostic groups, organisations, or electronic medical records. The system has been standard practice since 2012 in epilepsy outpatient clinics and subsequently in eight other diagnostic groups. Experiences from the nine telePRO implementations have shown an impact on the organisation of patient care, since 48 % of the epilepsy respondents did not need further contact with the clinic other than the PRO itself. This could indicate the need for a reorganisation of conventional care in the healthcare system. Finally, based on our experiences, we recommend use of telePRO for patients with chronic diseases with many consecutive contacts, where PRO is essential for clinical evaluation. In the implementation process for new patient groups, involvement of patients as well as frontline clinicians and administrative leaders is essential.
